# Supplementary material for: Sponge exhalent seawater contains a unique chemical profile of dissolved organic matter
Source: PeerJ. 2017 Jan 11;5:e2870. doi: 10.7717/peerj.2870 (PMC5234435; doi:10.7717/peerj.2870)
Supplement: Table S1 [file peerj-05-2870-s001.docx]

**Supplemental Material:**

**Sponge exhalent seawater contains a unique chemical profile of dissolved organic matter**

Cara L. Fiore^1,*^, Christopher J. Freeman^2^, Elizabeth B. Kujawinski^1^

^1^Marine Chemistry and Geochemistry, Woods Hole Oceanographic Institution, Woods Hole, MA 02543, U.S.A.

^2^ Smithsonian Marine Station, Fort Pierce, FL 34949, U.S.A

^*^Current address: Biology Department, Appalachian State University, Boone, NC 28607, U.S.A.

Table S1. Statistical results from correlations between metabolite concentration and sample type (inhalant, exhalent, off reef seawater) for targeted metabolomics data. These data were used to overlay vectors onto the nMDS plot in Figure 5. Bolded p-values indicate statistical significance and the sample type that the metabolite was significantly correlated with is shown.

| **Compound** | **r^2^** | **p-value** | **Sample type** |
| --- | --- | --- | --- |
| Tryptophan | 0.76 | **0.00** | Off reef |
| Kynurenine | 0.65 | **0.01** | Off reef |
| Phenylalanine | 0.71 | **0.00** | Off reef |
| Tryptamine | 0.67 | **0.00** | Off reef |
| Tyrosine | 0.81 | **0.00** | Off reef |
| 4-hydroxybenzoic acid | 0.56 | **0.02** | Inhalant |
| 4-aminobenzoic acid | 0.12 | 0.46 |  |
| Glycerol-3-phosphate | 0.31 | 0.12 |  |
| MTA | 0.12 | 0.49 |  |
| Adenosine | 0.56 | **0.02** | Exhalent |
| Guanosine | 0.60 | **0.01** | Exhalent |
| Inosine | 0.35 | 0.09 |  |
| Xanthosine | 0.77 | **0.01** | Exhalent |
| Caffeine | 0.68 | **0.01** | Off reef |
| Pantothenic acid | 0.05 | 0.73 |  |
| Riboflavin | 0.57 | **0.01** | Exhalent |
